# Nearly Complete Genome Sequence of Human Influenza Virus Strain A/Almaty/6327/2014 (H1N1) from Central Asia

**DOI:** 10.1128/MRA.00527-19

**Published:** 2019-11-21

**Authors:** Kobey Karamendin, Aidyn Kydyrmanov, Saule Asanova, Elizaveta Khan, Klara Daulbayeva, Yermukhammet Kasymbekov, Kainar Zhumatov, Nazgul Asanova, Marat Sayatov

**Affiliations:** a Laboratory of Viral Ecology, Institute of Microbiology and Virology, Almaty, Kazakhstan; b Children’s Municipal Hospital No. 2, Almaty, Kazakhstan; KU Leuven

## Abstract

An influenza virus strain, A/Almaty/6327/2014 (H1N1), was isolated in Almaty (in southeastern Kazakhstan) during a human population surveillance study in 2014. Here, we present the nearly complete genome sequence of this epidemic strain that was compared to the postpandemic variants of A(H1N1)pdm09.

## ANNOUNCEMENT

Influenza A viruses (IAV) belong to the genus Alphainfluenzavirus in the *Orthomyxoviridae* family and cause seasonal diseases with high morbidity and mortality in 5 to 10% of the world’s human population (https://www.who.int/biologicals/vaccines/influenza/en/). Human IAV are constantly changing, and it is important to follow the molecular evolution of the circulating strains to regularly update the composition of the influenza A vaccine.

During a seasonal influenza epidemic in Almaty, Kazakhstan, the strain A/Almaty/6327/2014 (H1N1) was isolated from nasopharyngeal swabs from patients with clinical symptoms of a respiratory disease. The virus was isolated by the inoculation of samples into 10-day-old embryonated chicken eggs with 48 h of incubation at 37°С (1). A hemagglutination test with chicken erythrocytes was performed to detect the virus. Viral RNA was extracted using the QIAamp viral RNA minikit (Qiagen), and its sequence was determined by next-generation sequencing using the NEBNext RNA sequencing kit along with the rRNA depletion kit (NEB, USA). Paired-end sequencing of multiple pooled samples was performed on an Illumina MiSeq instrument, using the MiSeq reagent kit v2 (Illumina). In total, approximately 914,000 raw sequencing reads per sample were obtained, with a mean length of 250 nucleotides per read. Sequencing quality analysis was performed using FastQC (2). Sequence data were trimmed and mapped against the 8 segments of the reference strain A/California/7/2009 (H1N1)pdm09 (GenBank accession numbers CY121680 to CY121687) using the Geneious 11.0 software (Biomatters), with default parameters. The final assembly of A/Almaty/6327/2014 (H1N1), obtained with Geneious 11.0, was 13,165 nucleotides in length, with a mean coverage of 8,158-fold.

To fill the gaps found in the neuraminidase (NA) and nucleoprotein (NP) gene sequences, these genes were simultaneously amplified with reverse transcription-PCR (RT-PCR) using the MBTuni-12 and MBTuni-13 primers (3). The NA and NP PCR gene products were excised from the gel, purified using a quick gel extraction kit (Invitrogen, Germany), and further Sanger sequenced on an ABI 3500 DNA analyzer using the BigDye Terminator 3.1 cycle sequencing kit (ABI, USA) with the same primers as those used in RT-PCR. The size of each obtained viral segment is shown in Table 1. A phylogenetic tree at the nucleotide level for the hemagglutinin gene was constructed using the neighbor-joining method and the Tamura-Nei model (4) in MEGA 7.0 (5).

**TABLE 1:** Comparison of the nucleotide sequences of all genes of the Kazakh strain with the genetically most closely related strains in GenBank

Gene or segment	Size (nucleotides)	GC content (%)	Most closely related strain	Identity at nucleotide level (%)	GenBank accession no.
PB2	2,282	44.04	A/New York/WC-LVD-14-003/2014 (H1N1)	99.74	CY189072
PB1	2,266	41.53	A/Porto Alegre/LACENRS-1573/2013 (H1N1)	99.69	KY925344
PA	2,207	43.36	A/Santa Cruz do Sul/LACENRS-1560/2013 (H1N1)	99.77	KY925663
HA	1,762	40.92	A/Porto Alegre/LACENRS-3653/2013 (H1N1)	99.38	KY926272
NP	1,522	45.40	A/Montenegro/LACENRS-2312/2013 (H1N1)	99.47	KY926177
NA	1,370	42.41	A/Fukuoka/DS4-50/2014 (H1N1)	99.64	LC409131
M	927	47.36	A/Rosario do Sul/LACENRS-1832/2013 (H1N1)	99.68	KY925132
NS	829	43.91	A/Germany/18909686/2015 (H1N1)	99.76	MK159112

Sequence analyses revealed that all genes of the isolated virus were similar to genes of pandemic A(H1N1)pdm09 or closely related strains, but had some particular signatures. Two substitutions, K163Q and S185T, were detected in the hemagglutinin Sa and Sb antigenic domains, which have been previously shown to potentially lead to altered antigenicity resulting in the escape from neutralizing antibodies (6). Four mutations were found (L191I, V199A, A73T, and D222G), differentiating the Kazakh strain from cocirculating postpandemic strains of 2013 to 2015. Interestingly, these 4 mutations (A73T and D222G located in domains Cb and Ca2, respectively) had been previously described in North America in 2009 and 2010.

**FIG 1:**
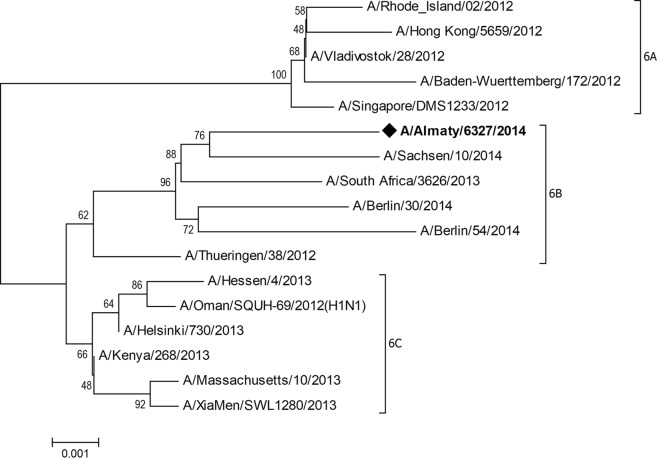
Phylogenetic tree of the HA gene of A/H1N1 viruses of clade 6 circulated around the globe.

Based on the lack of the specific substitution H274Y, we speculate that the Kazakh isolate was sensitive to neuraminidase inhibitor drugs (7), but the neuraminidase gene had 12 other mutations. The remaining genes of A/Almaty/6327/2014 (H1N1) showed evolutionary changes similar to those of strains isolated in 2013 to 2015 and which circulated around the globe (8–10). Phylogenetic analysis of the HA sequence of the A/Almaty/6327/2014 (H1N1) isolate showed that it belonged to clade 6B, which evolved after the 2009 global pandemic (Fig. 1).

### Data availability.

The 8 nearly complete genome segments are available under GenBank accession numbers MK788324 to MK788331. Raw sequence reads were deposited under BioProject number PRJNA532809.
